# Supplementary material for: Percutaneous Irreversible Electroporation for Treatment of Small Hepatocellular Carcinoma Invisible on Unenhanced CT: A Novel Combined Strategy with Prior Transarterial Tumor Marking
Source: Cancers (Basel). 2021 Apr 22;13(9):2021. doi: 10.3390/cancers13092021 (PMC8122342; doi:10.3390/cancers13092021)
Supplement: Supplementary file 1 [file cancers-13-02021-s001.zip › cancers-1174335-SI.pdf]

cancers

MDPI

Supplementary Material: Percutaneous Irreversible Electroporation for Treatment of Small Hepatocellular Carcinoma Invisible on Unenhanced CT: A Novel Combined Strategy with Prior Transarterial Tumor Marking

Feng Pan, Thuy D. Do, Dominik F. Vollherbst, Philippe L. Pereira, Götz M. Richter, Michael Faerber, Karl H. Weiss, Arianeb Mehrabi, Hans U. Kauczor and Christof M. Sommer

Electronic Supplement-1. Parameters of Trans-microcatheter Contrast-enhanced Cone-beam CT

Major acquisition parameters of cone-beam CT included an 8 s rotational acquisition generating 396 projections with an angular step of 0.5° for a total coverage of 200° with a pulse length of 5 ms and a dose per frame of 0.36 μGy. The contrast material injection protocol was modified as follows: injection speed of 2 ml/s, injection volume of 28 ml (pure or diluted contrast material) and an X-ray delay of 6 s. Cone-beam CT images were reconstructed as MPR images (transverse and coronal slice orientation; soft tissue and bone window preset) with a slice thickness of 1 mm and an increment of 0.6 mm, and 3D rendering images. Besides, the supplying arterial branch of the target HCC was manually marked by selecting two or more artery points on axial images and automatically identified using the "embolization guidance" module in syngo X Workplace (VB21N P05 Rev.7, Siemens Healthineers, Erlangen, Germany). Afterwards, the 3D rendering image with marked supplying arterial branch of target-HCC was reconstructed as a reference to assist with micro-catheterization (Figure 1D).

Electronic Supplement-2. Intra-observer and Inter-Observer Agreements of the Hounsfield scale Measurements

		Intra-Observer Agreement		Inter-Observer Agreement
		Observer 1	Observer 2	Observer 1 vs. Observer 2*
Pre-marking CT	ROIs (mm^2)	3.3 (−8.9, 15.4)	0.6 (−14.9, 16.1)	−0.5 (−8.0, 6.9)
	Hounsfield scale of the target-HCCs (HU)	1.5 (−1.9, 4.9)	−0.5 (−1.1, 0.0)	−0.3 (−1.4, 0.8)
	Hounsfield scale of the surrounding normal liver tissue (HU)	−0.6 (−1.9, 0.8)	−1.4 (−3.4, 0.7)	0.3 (−0.8, 1.5)
	Hounsfield scale of the peripheral normal liver tissue (HU)	−1.5 (−4.7, 1.7)	0.7 (−1.1, 2.6)	0.4 (−1.3, 2.2)
	Standard deviation for Hounsfield scale of the paraspinal muscle (HU)	0.3 (−1.4, 2.0)	−0.9 (−2.4, 0.6)	−1.4 (−3.1, 0.3)
Post-marking CT	ROIs (mm^2)	−0.7 (−11.5, 10.1)	−5.4 (−15.9, 5.0)	4.4 (−3.7, 12.5)
	Hounsfield scale of the target-HCCs (HU)	2.1 (−2.1, 6.4)	0.9 (−3.2, 5.0)	−2.1 (−8.1, 4.0)
	Hounsfield scale of the surrounding normal liver tissue (HU)	1.1 (−5.9, 8.1)	3.8 (−3.1, 10.7)	1.6 (−4.9, 8.1)
	Hounsfield scale of the peripheral normal liver tissue (HU)	1.3 (−1.3, 3.8)	−1.0 (−4.1, 2.0)	−0.6 (−1.6, 0.4)
	Standard deviation for Hounsfield scale of the paraspinal muscle (HU)	0.0 (−1.5, 1.6)	0.6 (−1.5, 2.8)	0.2 (−0.9, 1.3)

The intra- and inter-observer agreements were presented as mean difference (95% confidence interval). * Calculated from mean values of Reading 1 and Reading 2 by Observer 1 and Observer 2, respectively.
